# Endothelial cell-selective adhesion molecule deficiency exhibits increased pulmonary vascular resistance due to impaired endothelial nitric oxide signaling

**DOI:** 10.1152/ajpheart.00593.2024

**Published:** 2024-12-31

**Authors:** Vadym Buncha, Liwei Lang, Katie Anne Fopiano, Daria V. Ilatovskaya, Gaston Kapuku, Alexander D. Verin, Zsolt Bagi

**Affiliations:** 1 Department of Physiology, Medical College of Georgia, Augusta University, Augusta, Georgia, United States; 2 Department of Medicine, Georgia Prevention Institute, Medical College of Georgia, Augusta University, Augusta, Georgia, United States; 3 Vascular Biology Center, Medical College of Georgia, Augusta University, Augusta, Georgia, United States

**Keywords:** ESAM, nitric oxide synthase, pulmonary artery, pulmonary vascular resistance

## Abstract

Endothelial cell-selective adhesion molecule (ESAM) is a member of tight junction molecules, highly abundant in the heart and the lung, and plays a role in regulating endothelial cell permeability. We previously reported that mice with genetic ESAM deficiency (*ESAM^−/−^*) exhibit coronary microvascular dysfunction leading to the development of left ventricular diastolic dysfunction. Here, we hypothesize that *ESAM^−/−^* mice display impairments in the pulmonary vasculature, affecting the overall pulmonary vascular resistance (PVR). We utilized *ESAM^−/−^* mice and employed isolated, ventilated, and perfused whole lung preparation to assess PVR independently of cardiac function. PVR was assessed in response to stepwise increases in flow, and also in response to perfusion of the endothelium-dependent agonist, bradykinin, the thromboxane analog, U46619, and the nitric oxide (NO) donor sodium nitroprusside (SNP). We found that PVR, at every applied flow rate, is significantly elevated in *ESAM^−/−^* mice compared with WT mice. Bradykinin-induced reduction in PVR and U46619-induced increase in PVR were both diminished in *ESAM^−/−^* mice, whereas SNP-induced responses were similar in wild-type (WT) and *ESAM^−/−^* mice. Inhibition of NO synthase with *N*(ω)-nitro-l-arginine methyl ester increased agonist-induced PVR in WT but not in *ESAM^−/−^* mice. Pulmonary arteries isolated from *ESAM^−/−^* mice exhibited a reduced level of phospho-Ser473-Akt and phospho-Ser1177-eNOS. Furthermore, in human lung microvascular endothelial cells cultured under flow conditions, we found that siRNA-mediated knockdown of ESAM impaired fluid shear stress-induced endothelial cell alignment. Thus, we suggest that ESAM plays an important role in the endothelium-dependent, flow/shear stress- and vasoactive agonist-stimulated, and NO-mediated maintenance of PVR in mice.

## NEW & NOTEWORTHY

Our study reveals a novel role for ESAM in contributing to the maintenance of pulmonary vascular resistance under normal physiological conditions. Employing mice with global genetic deficiency of ESAM and using isolated whole lung preparation, we show significant impairments in nitric oxide-mediated pulmonary artery function. In vitro cell culture studies demonstrate impaired fluid shear stress-induced cell alignment in human lung endothelial cells after siRNA-mediated ESAM knockdown.

## INTRODUCTION

The endothelial cell-selective adhesion molecule (ESAM) is an immunoglobulin-like cell adhesion molecule and a member of the family of endothelial cell junctional adhesion molecules (1). ESAM is abundantly expressed in the heart and the lung with lower expression levels detected in other organs, such as the kidney and skin (1). Functionally, ESAM stabilizes endothelial cell junctions, thereby contributing to the control of endothelial permeability (2-6). A prior study has shown that genetic inactivation of ESAM enhances vascular permeability in the lung, whereas the heart, skin, and brain vasculature were not affected (7). ESAM has also been demonstrated to play a critical role in the angiogenic processes (8, 9). We recently reported that mice with genetic ESAM deficiency have an impaired angiogenic response in the heart and exhibit coronary microvascular rarefaction and left ventricle (LV) diastolic dysfunction (10). Although lung permeability remained unchanged under basal conditions, we noted a significant development of pulmonary edema in ESAM knockout mice following a hemodynamic challenge (10). The role of ESAM in contributing to pulmonary vascular function remains largely unexplored.

It is known that LV diastolic dysfunction can result in structural remodeling of the pulmonary circulation (11-13). Increased left-side filling pressure is often associated with excessive pulmonary vasoconstriction with or without vascular remodeling leading to a persistent increase in pulmonary vascular resistance. Increased pulmonary vascular resistance represents an important prognostic clinical parameter, particularly in the context of pulmonary prehypertension among patients with diastolic heart failure, also known as heart failure with preserved ejection fraction, or HFpEF (14-16). Patients with HFpEF having pulmonary complications develop more severe symptoms and suffer significant exercise intolerance, more frequent hospitalizations, and reduced survival (11). In patients with hypertension, pulmonary arterial pressure increases in correlation with pulmonary capillary wedge pressure (PCWP), which estimates left atrial filling pressure (15). However, even after adjusting for PCWP, individuals with LV diastolic dysfunction still exhibit higher pulmonary arterial pressure readings (15). These clinical observations underscore the important interrelationship between cardiac and pulmonary function, both in health and disease, and also highlight the challenges of studying pulmonary vascular function independently of cardiac influences.

The role of ESAM in influencing pulmonary vascular resistance, even independently of LV diastolic dysfunction in the ESAM knockout mice, along with its underlying molecular mechanisms, remains unclear. To investigate this, we used homozygous genetic deletion of ESAM (*ESAM^−/−^*) in our study. We employed isolated, ventilated, and perfused lung preparations to evaluate ESAM’s impact on pulmonary vascular function excluding in vivo cardiac influences. Our findings indicate that ESAM knockout mice exhibit elevated pulmonary vascular resistance due to pulmonary arterial endothelial dysfunction, likely to be mediated by impaired mechanosensitive activation of nitric oxide (NO) signaling pathways.

## METHODS

### Animals

The work involving experimental animals was conducted under the protocol approved by the Institutional Animal Care and Use Committee at the Medical College of Georgia, Augusta University. All experimental animal procedures performed in this study were in compliance with the European Convention for the Protection of Vertebrate Animals used for Experimental and other Scientific Purposes. Experiments were carried out in 16–20-wk old *ESAM^−/−^* (Strain No.: JAX:005862, Jackson Laboratory) and C57BL/6J wild-type (WT) littermates, both males and females. The mice were housed in the animal care facility with a 12-h light:dark cycle; access to rodent chow and tap water was provided ad libitum.

### Ultrasound Doppler Assessment of Pulmonary Artery Hemodynamics

Animals were anesthetized with 2% isoflurane and the ultrasound analysis was performed using a high-frequency ultrasound system (VEVO 3100 and MX250 transducer, VisualSonics, Toronto, ON, Canada). The pulmonary artery was visualized from the long parasternal axis, appearing as a vessel perpendicular to the aorta. A pulse-wave Doppler measurement gate was positioned near the right ventricular outward tract in a point with the highest blood velocity. The pulmonary artery acceleration time (PAT) to ejection time (PET) ratio (PAT/PET) was then calculated using VevoLab 4.5.1.

### Right Ventricle Hypertrophy Assessment (Fulton Index)

The heart was carefully dissected from the animal and placed in a Petri dish containing ice-cold, Ca^2+^-free physiological saline solution (PSS). Using fine microscissors, the atria were removed, and the right ventricle (RV) was carefully isolated from the heart. The RV, the remaining left ventricle (LV) and the septum (S) were then weighed using an analytical scale (0.0001 g). Right ventricular hypertrophy (Fulton’s index) was calculated using the formula: FI=RV∕(LV+S).

### Ex Vivo Lung Perfusion

**Figure 1.**
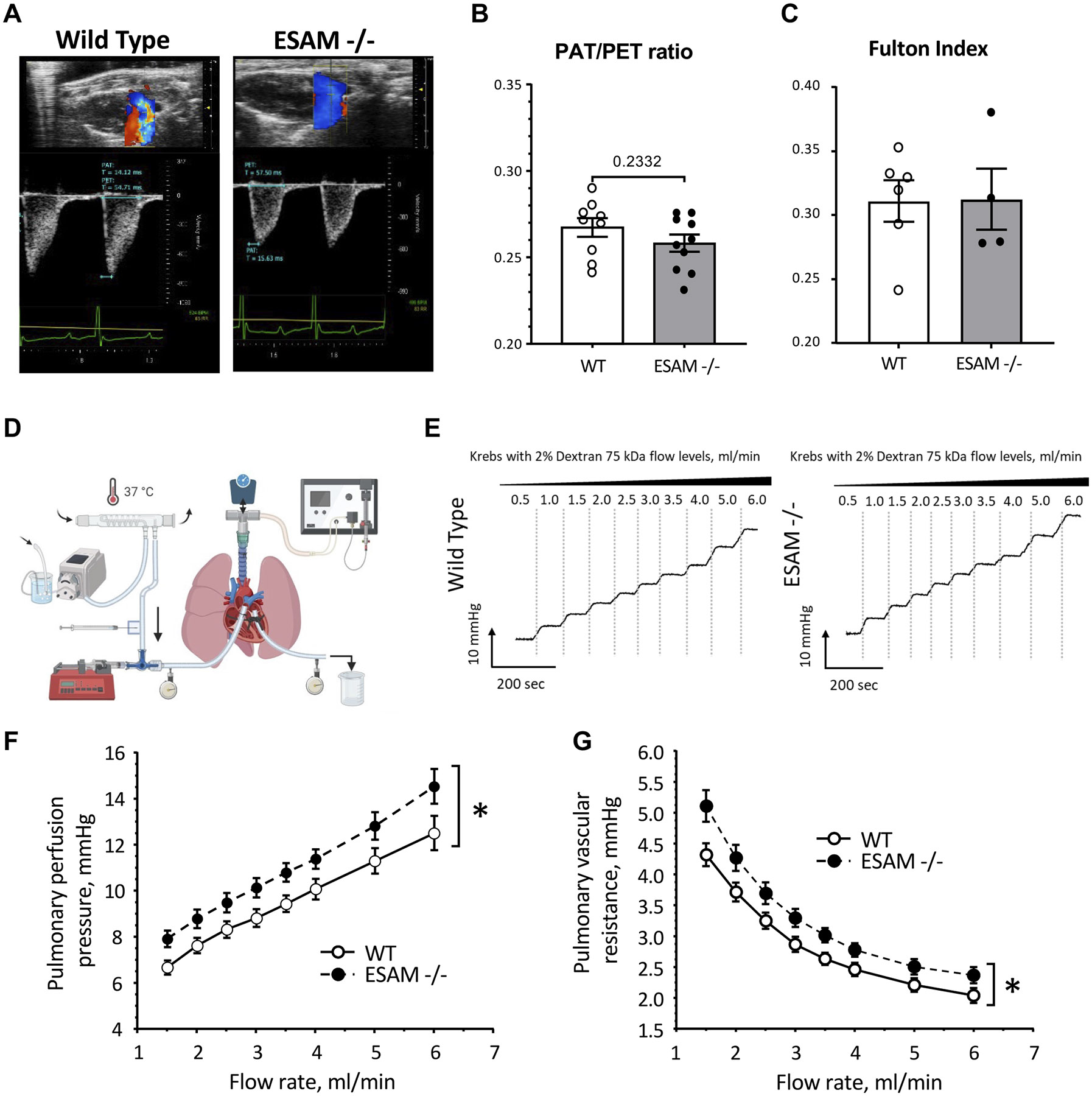
Increased pulmonary vascular resistance in *ESAM^−/−^* mice. Representative ultrasound color Doppler images in parasternal long axis mode and traces of pulmonary artery blood flow velocity (*A*), with summary data of calculated pulmonary acceleration time (PAT) to pulmonary ejection time (PET) ratio (PAT/PET; *B*) in WT (*n* = 9) and *ESAM^−/−^* (*n* = 10). *C*: summary data of Fulton’s index in WT (*n* = 6) and *ESAM^−/−^* (*n* = 4). *D*: schematic representation of the experimental setup for the isolated, perfused, and ventilated whole lung preparation. *E*: representative traces for pulmonary vascular perfusion pressure measurement in response to stepwise increases in perfusion flow rates in WT and *ESAM^−/−^* mice. Summary data of pulmonary perfusion pressure (*F*) and calculated pulmonary vascular resistance (*G*) in response to stepwise increases in perfusion flow in WT (*n* = 6) and *ESAM^−/−^* (*n* = 6) mice. Data presented as means ± SE and analyzed using *t* test with Welsh’s correction (*B* and *C*) or two-way ANOVA with repeated measures (*F* and *G*); *P < 0.05. ESAM, endothelial cell-selective adhesion molecule; WT, wild-type.

Mice were anesthetized with 3% isoflurane. The trachea was cannulated and immediately ventilated [respiratory rate 140 breaths/min with a tidal volume of 250 μL and positive end-expiratory pressure (PEEP) 1 cmH_2_O] using a small animal ventilator (RWD, VentStar R315) with 3% isoflurane mixed with room air. Sodium heparin (100 units per 100 g body wt) was injected into the vena cava and allowed to circulate for 1 min. Afterward, the animal was euthanized via exsanguination following transection of the abdominal vena cava. The thoracic cavity was opened, and a cannula was inserted into the pulmonary artery and secured with a ligature. An incision in the left ventricle anterior wall was made using scissors and perfusion with Ca^2+^-free Krebs–Henseleit solution containing 2% Dextran (MW 75000, Thermo Scientific, Cat. No. J60989.22) was initiated at a constant flow of 0.3 mL/min. The perfusion solution was filtered and pH was adjusted to 7.4±0.05; throughout the experiment, the temperature of the perfusate was maintained at 37°C, and aeration was provided with 5% CO_2_ and 95% O_2_. We used 95% O_2_ to facilitate oxygen delivery in the asanguinous preparation with limited oxygen-carrying capacity. The outflow cannula was inserted from the left ventricle and secured in the left atrium after which, the preparation was dissected from the rib cage and attached to an isolated lung system (Fig. 1A). At this point, the perfusion buffer was switched from Ca^2+^-free to the Ca^2+^-containing (2 mM) solution. The outflow cannula connected to the outflow reservoir was placed at the level of the ventricle, so the outflow pressure was controlled and maintained at the level of0 ± 0.2 mmHg.

### Assessment of Pulmonary Vascular Resistance in Isolated Lungs

In the experimental protocol, flow (Q) was established with a peristaltic pump (Masterflex Ismatec Reglo ICC Digital Pump, Model No.: 78001-80). The pulmonary arterial perfusion pressure (Pa) and the pulmonary venous perfusion pressure (Pv) were monitored, whereas the flow rate was changed in a stepwise manner (Fig. 1B). Pulmonary vascular resistance was calculated as PVR =(Pa−Pv)∕Q.

**Figure 2.**
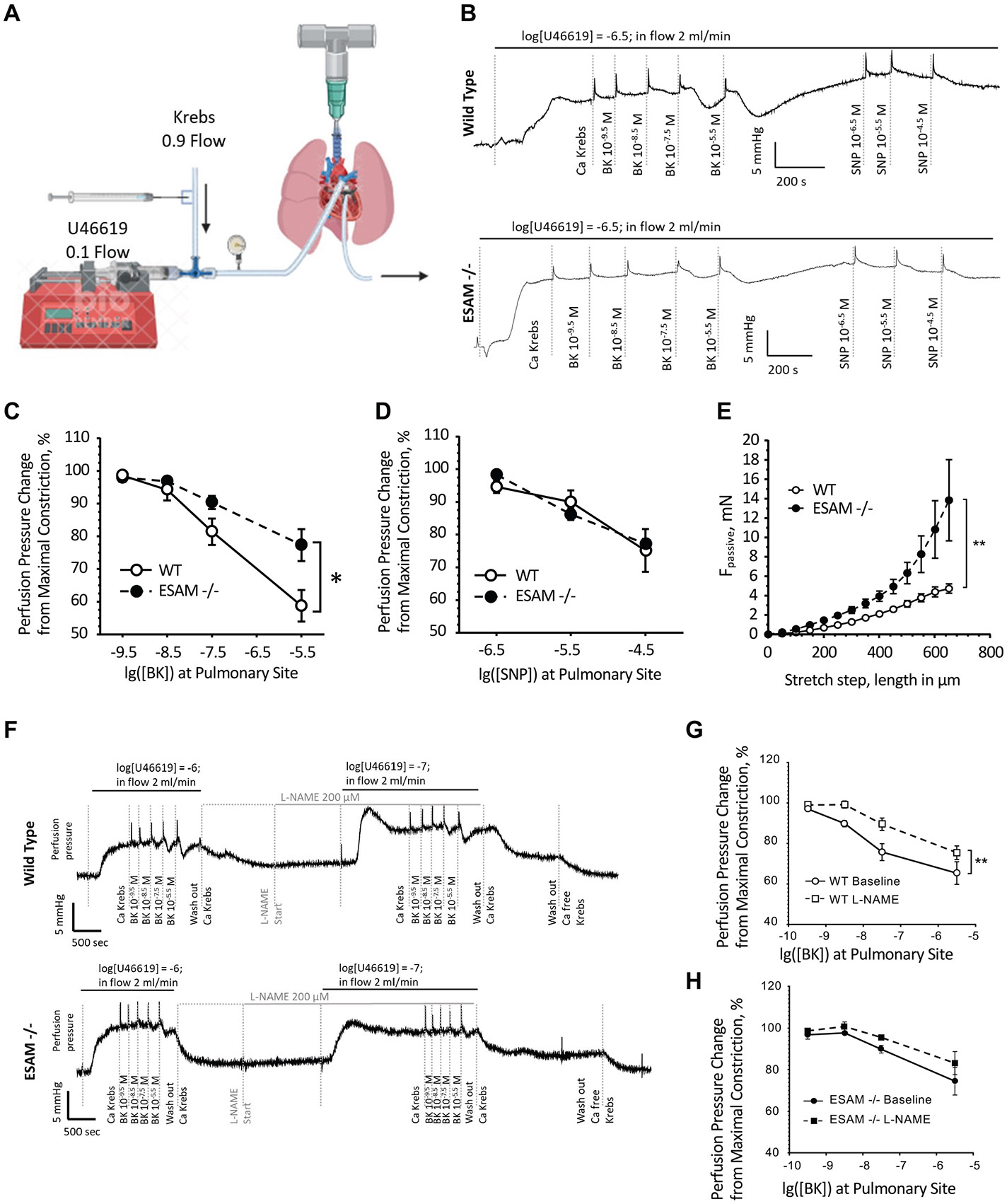
Impaired endothelium-dependent, agonist-stimulated reduction of PVR in *ESAM^−/−^* mice. A: schema for the ex vivo whole lung preparation setup used in drug administration experiments. Representative traces (*B*) and summary data of changes in perfusion pressure in response to injection of cumulative concentration of bradykinin (*C*) or sodium nitroprusside (SNP) (*D*) in WT (*n* = 6) and *ESAM^−/−^* (*n* = 6) mice. Note that a sudden increase in perfusion pressure occurs at the onset of drug injections, which is considered an artifact. *E*: summary data for the generated vascular passive force (*F*_Passive_) in response to stepwise stretching of isolated pulmonary arteries (WT, *n* = 10 vessels isolated from 4 animals; *ESAM^−/−^* *n* = 5 vessels isolated from 2 animals) in calcium-free PSS. Representative traces (*F*) and summary data showing the bradykinin-induced response in the absence or presence of NO synthase inhibitor, L-NAME in WT (*G*) and *ESAM^−/−^* (*H*) mice. Data are presented as means ± SE and analyzed using two-way ANOVA with repeated measures; *P < 0.05, **P < 0.01. ESAM, endothelial cell-selective adhesion molecule; L-NAME, *N*(ω)-nitro-l-arginine methyl ester; NO, nitric oxide; PSS, physiological saline solution; PVR, pulmonary vascular resistance; WT, wild-type.

An additional line was added to the circuit for the constant bolus injection of the preconstrictor, U46619, as presented in Fig. 2A. Ca^2+^-containing Krebs solution with U46619 was infused into the perfusate stream to maintain the overall concentration of 10 × 10^−6^ M and flow rate 2 mL/min. When perfusion pressure stabilized, 50 μL of Ca^2+^-containing Krebs solution was injected as a negative control for the perfusion pressure change. Following, 50 μL bolus injections of increasing concentrations of bradykinin (BK, 10^−9.5^, 10^−8.5^, 10^−7.5^, and 10^−5.5^ M) or sodium nitroprusside (SNP, 10^−6.5^, 10^−5.5^, and 10^−4.5^ M) were performed through the injection port, and the decrease in perfusion pressure was monitored and interpreted as a reflection of pulmonary vasculature relaxation (Fig. 2B). The relative response was calculated as the percent of perfusion pressure drop from the U46619-induced increase in perfusion pressure.

To further investigate whether the BK-induced endothelium-dependent response is mediated by NO, the BK dose-dependent response was tested in the presence of the NO synthase inhibitor *N*(ω)-nitro-l-arginine methyl ester (L-NAME, 200 μM; Fig. 2F). In this experiment, the dose-dependent response to BK was initially tested on a background of U46619 (10^−6^ M) as described in Assessment of Pulmonary Vascular Resistance in Isolated Lungs. Following, the preconstrictor was washed out with Ca^2+^-containing Krebs solution. Once the perfusion pressure stabilized at baseline, the perfusate was switched to a Ca^2+^-containing Krebs solution with L-NAME, and the preparation was incubated for 10 min. A second dose-dependent response to BK was tested on a background of U46619 (10^−7^ M). The concentration of U46619 was lowered to prevent excessive constriction and maintain perfusion pressure on a level similar to the first part of the experiment. Finally, the preparation was washed by perfusion with Ca^2+^-free Krebs. The relative response was calculated as the percent of perfusion pressure drop from the U46619-induced increase in perfusion pressure to Ca^2+^-free baseline of perfusion pressure.

To assess NO synthase-dependent response to U46619 preconstriction, Ca^2+^-containing Krebs solution was maintained at a 2 mL/min flow rate when two repetitive bolus injections of U46619 (50 μL, 1 × 10^−6.5^ M final concentration) were performed to stimulate vessel constriction and an increase in perfusion pressure, before and after the incubation with NO synthase inhibitor *N*(ω)-nitro-l-arginine methyl ester (L-NAME, 200 mM, incubation time 10 min before the second injection, started when response after the first injection returned to baseline) (Fig. 2E). Peak increases of perfusion pressure were compared and analyzed.

### Wire Myography of Isolated Pulmonary Artery

Third-order pulmonary arteries were freshly isolated from the left lobe of mouse lung and mounted on multi myograph system (Model 610, DMT) in Ca^2+^-free physiological saline solution (PSS) containing NaCl (118 mM), KCl (4.7 mM), MgSO_4_·7H_2_O (1.2 mM), KH_2_PO_4_ (1.2 mM), Dextrose (5.5 mM), NaHCO_3_ (25 mM), adjusted to pH 7.4, constantly aerated with gas mixture of 95% O_2_ and 5% CO_2_, at 37°C. Tension acquisition was performed with LabChart 8.0 (v8.1.13, ADInstruments). In the Ca^2+^-free PSS, passive force (*F*_Passive_) development was recorded in response to vessel stretch, which was achieved by incrementally increasing the distance between the jaws by 5 μm per step.

### Western Immunoblotting and Coimmunoprecipitation

Pulmonary arteries were homogenized in tissue protein extraction reagent (T-PER, Cat. No. 78510, Thermo Fisher) buffer mixed with 1% protease inhibitor cocktail (P8340, Sigma) and phosphatase inhibitors. Protein concentration was measured with BCA assay (Cat. No. 23227, Thermo Scientific). After blotting, membranes (Hybond-P, GE Healthcare) were probed with pS1177-endothelial nitric oxide synthase (eNOS) primary antibody (1:500, Cat. No. PA5-17917, Invitrogen), pT308-Akt antibody (1:1,000, Cat. No. 2965, Cell Signaling Technologies), pS473-Akt (1:1,000, Cat. No. 4060, Cell Signaling Technologies), total-eNOS (1:1,000, Cat. No. 32027, Cell Signaling Technologies), and total-Akt (1:1,000, Cat. No. 4691, Cell Signaling Technologies) followed by incubation with horseradish peroxidase (HRP)-linked secondary antibody (anti-rabbit 1:2,000, Cat. No. 7074S, Cell Signaling Technologies). Enhanced chemiluminescence was visualized autoradiographically by the Fluochem E system and analyzed in ImageJ 1.54f. Protein expression was normalized to the HSP90 protein expression (Cat. No. 60318-1-IG, Thermo Fisher).

Coimmunoprecipitation between ESAM and VE-cadherin as well as ESAM and platelet endothelial cell adhesion molecule (PECAM)-1 were performed using Protein G magnetic beads following the manufacturer instructions (70024, Cell Signaling Technology, Danvers, MA). In brief, 200 μL lysates of endothelial cells (1 μg/μL) were incubated with rabbit polyclonal ESAM antibody (Abcam, Cat. No. ab74777, 1:100) overnight at 4°C with rotation. The cell lysate and antibody mixture were then transferred to a tube containing 20 μL of prewashed Protein G magnetic beads and incubated at room temperature with rotation for 20 min. The beads were then pelleted and washed using a magnetic separation rack, and the immunoprecipitated proteins were collected for Western blotting detection using anti-VE-cadherin or PECAM-1 antibodies.

### Immunocytochemistry

For immunocytochemistry human lung microvascular endothelial cells (HLMVECs) were grown onto collagen-coated glass coverslips and subsequently fixed with 4% paraformaldehyde. Unspecific antibody binding was blocked with 2% BSA in PBS for 1 h. Cells were incubated with goat antihuman ESAM IgG antibody (1:200, Cat. No. AF2688, R&D) or VE-cadherin (1:200, Cat. No. D87F2, Cell Signaling) overnight at 4°C. Alexa Fluor 647 conjugated donkey anti-goat IgG (1:250, Cat. No. ab150131, Abcam) and Alexa Fluor 488 conjugated donkey anti-rabbit IgG (1:250, Cat. No. SA5-10038, Invitrogen) were added for 1 h. DAPI (Vector Laboratories) was used for nuclear staining. Structured illumination microscopy (SIM-Apotome, AxioImagerM2, CarlZeiss) was used for immunofluorescence detection.

### Endothelial Cell Culture under Flow

Human lung microvascular endothelial cells (HLMVECs) were purchased from PromoCell (Cat. No. C-12281, Heidelberg, Germany). HLMVECs were cultured in endothelial cell growth medium 2 and used between passages 4 and 10. To knockdown ESAM expression, transfection of HLMVECs with ESAM small interfering RNA (siRNA, Dharmacon) was carried out using Lipofectamine RNAiMAX Transfection Reagent (Thermo Fisher Scientific). Nontargeting, scrambled siRNA was used as a control. Knockdown efficiency was tested with Western immunoblot (Fig. 4B). HLMVECs (1 × 10^5^ cells/slide) were seeded onto IbiTreat μ-slides I 0.6 or IbiTreat μ-slides I 0.4 (Cat. No. 50-305-753/6, Ibidi) and placed in an incubator at 37°C with 5.0% CO_2_. Wall shear stress (WSS) of 8 dyn/cm^2^ was applied to confluent HLMVECs using an Ibidi pump system (Cat. No. 10902, Ibidi, Germany) for 72 h. Phase contrast microscopy was used to collect images every 24 h. The ImageJ directionality plugin was used to measure cell orientation angle distribution relative to the flow direction.

### Statistical Analysis

All statistical analyses were performed using GraphPad Prism Software. Data comparisons between groups repeatedly over time were analyzed by two-way repeated-measures ANOVA followed by Tukey’s post hoc test for multiple comparisons or with two-tailed, unpaired Student’s *t* test (Western blots), as appropriate. Data are expressed as means ± SE with individual data points. *P* < 0.05 was considered statistically significant.

## RESULTS

### Pulmonary Vascular Resistance is Elevated in *ESAM^−/−^* Mice

First, noninvasively evaluated established indices of pulmonary hypertension and right ventricular morphology by measuring the pulmonary artery acceleration time (PAT) to ejection time (PET) ratio (PAT/PET), as well as calculating the Fulton index. We found a nonsignificant trend toward a reduced PAT/PET ratio in *ESAM^−/−^* mice (Fig. 1, A and B), and no changes were observed in the Fulton index compared with WT animals (Fig. 1C).

To exclude the in vivo influence of cardiac pump dysfunction, that we previously reported in *ESAM^−/−^* mice (10), we assessed pulmonary vascular resistance using an ex vivo perfused and ventilated lung preparation (Fig. 1D). This approach allowed us to measure changes in perfusion pressure under well-defined flow rate conditions and to calculate pulmonary vascular resistance (17). Although incrementally increasing the perfusion flow rate (Fig. 1E), we found that the perfusion pressure was significantly higher at each flow rate in *ESAM^−/−^* mice, when compared with wild-type controls (Fig. 1F). Accordingly, at each flow rate the calculated pulmonary vascular resistance was significantly elevated in the isolated lungs of *ESAM^−/−^* mice (Fig. 1G).

### Impaired Endothelium-Dependent Vasodilator Function in Pulmonary Artery of *ESAM^−/−^* Mice

To investigate the underlying mechanisms of increased pulmonary vascular resistance in *ESAM^−/−^*mice, we evaluated the effect of pharmacological agonists with known mechanisms of action in isolated lungs. At constant perfusion flow rate (2 mL/min) and in the presence of preconstrictor thromboxane receptor agonist U46619, the infusion of bradykinin, an endothelium-dependent agonist, elicited a dose-dependent decrease in pulmonary perfusion pressure, which was significantly reduced in the isolated lungs of *ESAM^−/−^* mice (Fig. 2, B and C). To determine whether the reduced pulmonary response in *ESAM^−/−^* mice was due to the impaired responsiveness of vascular smooth muscle cells we used the endothelium-independent vasodilator, NO donor, and sodium nitroprusside (SNP). SNP infusion induced a dose-dependent decrease in pulmonary perfusion pressure, which was, however, similar in the two groups (Fig. 2D).

In addition, passive force measurements of isolated pulmonary arteries (in a calcium-free solution) were performed through wire myography experiments. When subjected to stepwise stretching, pulmonary arteries isolated from *ESAM^−/−^* mice exhibited a significantly increased passive force, which may indicate an increase in vascular stiffness due to abnormal vascular wall remodeling (Fig. 2E).

### Impaired Activation of Pulmonary Vascular Endothelial NO Signaling in *ESAM^−/−^* Mice

**Figure 3.**
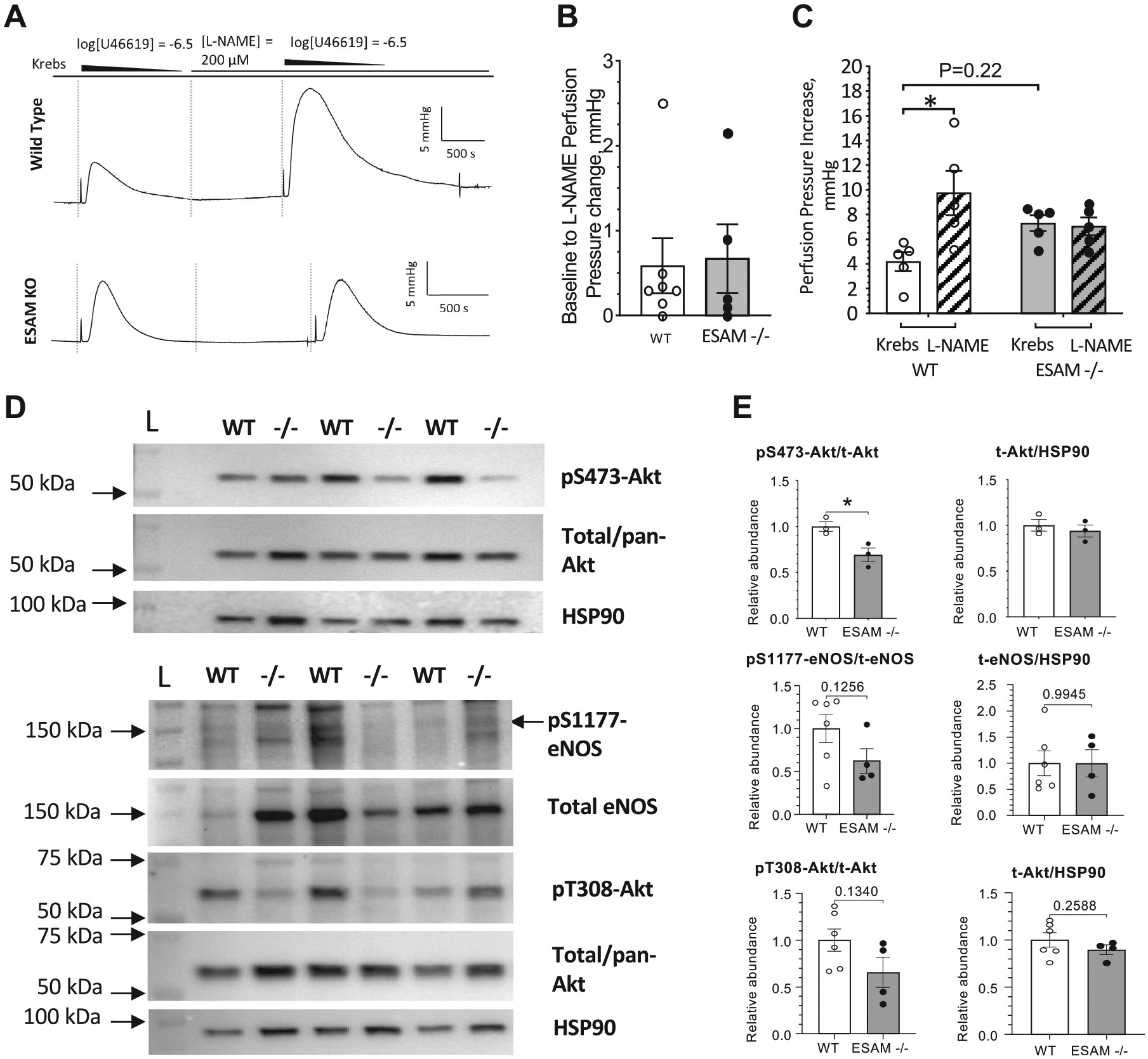
Impaired NO-mediated signaling in pulmonary arteries. Representative traces (*A*) and summary graph for basal (*B*) and U46619-induced changes in perfusion pressure (*C*), both in the absence and presence of L-NAME, in WT (*n* = 5) and *ESAM^−/−^* (*n* = 5) mice. Representative images of Western immunoblots for protein abundance of total eNOS and Akt and also pS1177-eNOS, pT308-Akt, and pS473-Akt in the pulmonary artery (*D* and *E*) isolated from WT and *ESAM^−/−^* mice. Summary graphs (*E*) representing the ratio of phosphorylated and total proteins (eNOS and Akt) and total proteins normalized for housekeeping HSP90 from the pulmonary artery. Data presented as means ± SE and analyzed with an ordinary two-way ANOVA, where the interaction between factors reaches significance (*P* = 0.0169), followed Tukey’s post hoc multiple comparisons (*C*) or unpaired *t* test with Welch’s correction (*B* and *E*); *P < 0.05. ESAM, endothelial cell-selective adhesion molecule; NO, nitric oxide; L-NAME, *N*(ω)-nitro-l-arginine methyl ester; WT.

Bradykinin causes vasodilation, in part, by activating endothelial NO signaling pathways (18). To determine the magnitude of the contribution of NO to pulmonary artery responsiveness, we measured basal and agonist-induced changes in pulmonary vascular resistance in the presence of NO synthesis inhibitor, L-NAME. First, we measured the effect of L-NAME on basal perfusion pressure under constant flow (2 mL/min) conditions. We found that L-NAME significantly increased perfusion pressure over baseline with a similar magnitude in wild-type and *ESAM^−/−^* mice (Figs. 2F and 3B). In contrast, in the presence of L-NAME, the BK-induced drop in PVR was significantly reduced in wild-type animals, whereas L-NAME did not affect BK responses in *ESAM^−/−^* mice (Fig. 2, G and H).

We moreover found that bolus injection of the vasoconstrictor U46619 caused a significantly larger increase in pulmonary perfusion pressure in wild-type mice, whereas L-NAME did not affect U46619-induced increases in pulmonary pressure in *ESAM^−/−^* mice (Fig. 3, A and C).

To estimate the activation level of NO signaling pathway we measured phospho-eNOS and phospho-Akt levels in isolated pulmonary arteries. The abundance of pS473-Akt was significantly lower in the pulmonary arteries of *ESAM^−/−^* mice compared with wild-type animals. Although there was no difference in total eNOS or Akt abundance in pulmonary arteries, the levels of pS1177-eNOS and pT308-Akt showed a trend toward reduced expression levels in pulmonary arteries of *ESAM^−/−^* mice (Fig. 3, D and E).

### Fluid Shear Stress-Dependent Endothelial Cell Alignment is Impaired after ESAM Knockdown in Cultured Human Lung Microvascular Endothelial Cells

**Figure 4.**
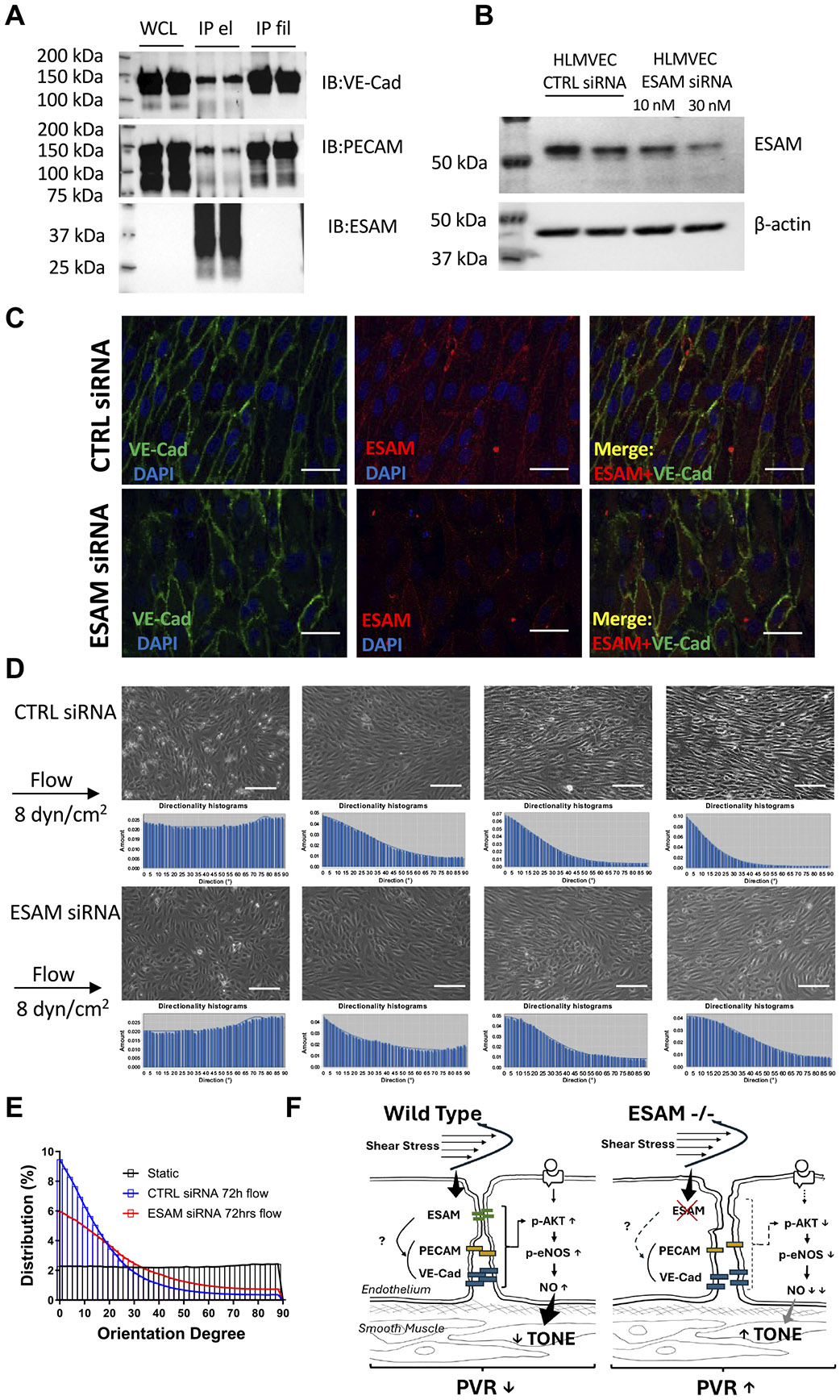
VE-cadherin and PECAM coimmunoprecipitate with endothelial cell-selective adhesion molecule (ESAM) while shear stress-induced endothelial cell alignment is impaired after ESAM knockdown. Representative western immunoblotting for ESAM/VE-cadherin and ESAM/PECAM complexes, as well as control for ESAM in cultured endothelial cells (*A*). Representative Western immunoblot (*B*) and immunocytochemical staining (*C*) show the effectiveness of ESAM knockdown in HMLVEC. VE-cadherin was used to co-label endothelial cells. Scale bar: 20 μm. Representative images (*D*) after 4, 24, 48, and 72 h of culture under flow and summary data (*E*) from triplicate experiments after 72 h of culture of HLMVEC with control (CTRL) or ESAM siRNA transfection. Scale bar: 100 μm. The ImageJ directionality plugin was used to measure cell orientation angle distribution relative to the flow direction. *F*: a schema depicts the proposed mechanisms through which ESAM contributes to agonist-induced and shear stress-stimulated and NO-mediated regulation of pulmonary vascular resistance (PVR). HLMVEC, human lung microvascular endothelial cell; IPel, immunoprecipitation eluate; IPfil, immunoprecipitation filtrate; NO, nitric oxide; WCL, whole cell lysates.

Using immunoprecipitation, we observed potential interactions between ESAM and VE-cadherin, as well as between ESAM and PECAM-1 in human lung microvascular endothelial cells (HLMVEC) cultured under flow conditions (Fig. 4A). Both VE-cadherin and PECAM-1 have been previously implicated in fluid shear stress mechanosensing and subsequent activation of NO signaling pathways (19). In response to laminal flow, endothelial cells align to the direction of flow and the degree of cell alignment indicates the cells’ ability to sense and respond to fluid shear stress, which directly relates to the magnitude of NO production (20). To examine the role of ESAM in this process HLMVEC were treated with ESAM-targeted siRNA or control siRNA, and then exposed to a steady laminar flow at a physiological shear stress level of 8 dyn/cm^2^ for 3 days, during which endothelial cell alignment was measured. Using Western blots and immunocytochemistry we have confirmed the reduced expression of ESAM in ESAM siRNA-treated HLMVECs (Fig. 4, B and C). Notably, we observed that endothelial cells with ESAM knockdown display a lower degree of orientation to the flow during the 3-day culture period compared with those transfected with control siRNA (Fig. 4, D and E).

## DISCUSSION

The results of this study demonstrate that *ESAM^−/−^* mice exhibit elevated pulmonary vascular resistance (PVR) and impaired NO-mediated, flow- and agonist-induced pulmonary vascular responses, which persist independent of cardiac pump dysfunction. *ESAM^−/−^* mice showed no signs of pulmonary hypertension, as assessed by PAT/PET measurements and the Fulton index. This highlighted the role of pulmonary artery endothelial dysfunction and impaired NO signaling underlying the early development of elevated PVR in *ESAM^−/−^* mice. Our study thus reveals a novel physiological role of ESAM in contributing to NO-mediated maintenance of PVR under normal conditions (Fig. 4F).

We recently reported that *ESAM^−/−^* mice develop LV diastolic dysfunction without signs of changes in pulmonary permeability under basal conditions (10). We observed that pulmonary edema developed only after the *ESAM^−/−^* mice were subjected to significant hemodynamic stress, induced by experimental volume overload and high blood pressure (10). Interestingly, these observed cardiovascular phenotypic changes in the *ESAM^−/−^* mice were reminiscent of that typically seen in patients with hypertension, who concurrently present with LV diastolic dysfunction and related pulmonary complications (21, 22). Clinical observations suggest that individuals with LV diastolic dysfunction often exhibit higher pulmonary arterial pressure readings even after adjusting for the increased left atrial filling pressures (15). It is recognized that patients with left-sided heart failure often develop elevated pulmonary pressures, which can lead to pulmonary prehypertension and eventually right-sided heart failure. This process is primarily due to the increased pressure in the left atrium, which translates into higher pulmonary venous pressure and subsequently causes increased resistance in the pulmonary vasculature (23). In the present study, we hypothesized that *ESAM^−/−^* mice display abnormal elevations in pulmonary vascular resistance, which persists independently of LV diastolic function.

First, we noninvasively evaluated established indices of pulmonary hypertension and right ventricular hypertrophy. A decreased PAT/PET ratio indicates higher pulmonary artery pressure and worse right ventricular function, whereas an increased Fulton index is a strong indicator of pulmonary hypertension (24). We found a nonsignificant trend toward a reduced PAT/PET ratio in *ESAM^−/−^* mice, and no changes were detected in the Fulton index. These observations indicated that *ESAM^−/−^* mice do not exhibit pulmonary hypertension and right ventricular dysfunction at the studied age (16-20 wk). Next, we employed ex vivo perfused lung preparation to evaluate pulmonary vascular function and PVR without the in vivo cardiac influences. Our findings reveal that *ESAM^−/−^* mice exhibit significantly higher perfusion pressure and PVR at each flow rate compared with wild-type controls. This indicates that normally ESAM contributes to the maintenance of PVR, whereas ESAM deficiency causes increases in PVR independent from that of cardiac pump dysfunction in *ESAM^−/−^* mice.

Increased PVR has been observed in various disease models that are associated with vascular endothelial dysfunction, supporting the link between endothelial health and impaired pulmonary vascular function (25). Here we show that bradykinin-induced, but not SNP-dependent vasodilation is impaired in *ESAM^−/−^* mice in isolated whole lung experiments, indicating an impairment in endothelial function, rather than altered vascular smooth muscle cell responsiveness, contributing to the increased PVR. It should be noted that when subjected to stepwise stretching, isolated pulmonary arteries from *ESAM^−/−^* mice exhibited increased passive force generation, which may indicate abnormal vascular wall remodeling. This change in pulmonary arterial biomechanics is similar to what we observed in the skeletal muscle resistance arteries of aged mice, which also exhibited impaired endothelial function (26).

Given the important roles of endothelium-derived NO in contributing to the regulation of pulmonary circulation, including its counteracting effects on pulmonary vasoconstriction, we investigated the role of ESAM in the activation of endothelial NO signaling pathways. In the presence of NO synthesis inhibitor L-NAME, bradykinin-induced reduction, as well as the vasoconstrictor U46619-induced increase in pulmonary perfusion pressure was greater in wild-type mice than in *ESAM^−/−^* mice, indicating an impairment of agonist-stimulated NO production or signaling in the latter. Studies have shown that Akt can directly phosphorylate eNOS and activate the enzyme, leading to an increased NO production (27, 28). Western blot analyses revealed a significant reduction in the abundance of pS473-Akt, a major regulator of eNOS activity, in pulmonary arteries of *ESAM^−/−^* mice. At the same time, a trend toward reduced levels of phosphorylated eNOS (pS1177-eNOS) and Akt (pT308-Akt) was observed in the pulmonary arteries of *ESAM^−/−^* mice. These data further support the hypothesis of compromised vascular endothelial NO signaling contributing to the observed elevated PVR in *ESAM^−/−^* mice.

The underlying mechanisms by which ESAM is linked to the activation of NO signaling pathways in pulmonary arteries remain incompletely understood. Previous studies have primarily focused on and have demonstrated the role of ESAM in regulating vascular permeability (6). Our previous study found that pulmonary edema develops in *ESAM^−/−^* mice only under hemodynamic stress (10). In a prior study, it was found that pulmonary barrier function in *ESAM^−/−^* mice was compromised only after administering a VE-cadherin-blocking antibody (7). These studies suggested that ESAM likely functions by interacting with other junctional proteins, including VE-cadherin. Interestingly, the putative endothelial wall shear stress mechanosensory complex comprises the interaction between several molecules, such as VE-cadherin and PECAM-1 (29). We have previously demonstrated that PECAM-1 directly senses temporal changes in fluid shear stress and stimulates NO production (30), and others have shown that VE-cadherin acts as an adaptor in this process (29). In the current study, we thus hypothesized that ESAM plays a role in maintaining NO-mediated PVR via interacting with other junctional adhesion molecules and, hence, modulating shear stress-dependent mechanosensitive mechanisms. In support of this scenario, we observed potential interactions between ESAM and VE-cadherin and also between ESAM and PECAM-1 in human lung microvascular endothelial cells cultured under flow conditions. We moreover found that after ESAM knockdown endothelial cells displayed an impaired alignment to the direction of flow. Although these results suggest a role of ESAM in endothelial cells’ ability to sense fluid shear stress and stimulate NO production, directly or indirectly by interacting with other mechanosensors, further studies are needed to elucidate the exact molecular mechanisms. For instance, a study by Ghimire et al. (31) suggested that a membrane-associated guanylate kinase, MAGI-1 (membrane-associated guanylate kinase, WW and PDZ domain-containing protein 1) regulates shear stress-mediated NO production through eNOS phosphorylation in endothelial cells. MAGI-1 is a multidomain adaptor protein, which binds to transmembrane, cytoskeletal, and signaling molecules, and is localized to tight junctions in epithelial cells (32). ESAM contains a C-terminal type I PDZ-binding domain and it was shown that MAGI-1 binds ESAM via its PDZ domain (33). It remains to be determined whether ESAM functions as part of the putative shear stress mechanosensory complex, in which ESAM interacts with other proteins, such as VE-cadherin, PECAM-1, and MAGI-1, to stimulate NO production.

Concerning the clinical and pathophysiological relevance of our study, it is important to note that, like many other adhesion molecules, the extracellular domain of ESAM can be cleaved and secreted into the bloodstream in a soluble form. Notable, in the Dallas Heart Study (involving 2,442 participants without prior cardiovascular disease) only soluble ESAM, but not soluble ICAM-1 or soluble VCAM-1, levels were associated with incidents of atherosclerotic and total cardiovascular disease (34). This observation aligns with available clinical data reporting increases in serum ESAM levels in patients with an enhanced risk for myocardial infarction, heart failure hospitalizations, and death (34, 35). It is intriguing to consider whether excessive cleavage of ESAM not only serves as a biomarker for cardiopulmonary pathologies, but whether the subsequent ESAM deficiency is also mechanistically linked to these pathological changes. Further research is needed to elucidate this possibility and potential mechanistic roles.

In summary, our study reveals a novel role for ESAM in maintaining pulmonary vascular resistance by facilitating endothelial NO signaling, as well as responses to vasoactive agonist stimulation and increased fluid shear stress. The elevated PVR observed in *ESAM^−/−^* mice is attributed to endothelial dysfunction, which occurs and persists independent from LV diastolic dysfunction. These findings also highlight the potential pathological role for ESAM deficiency, which via inducing LV diastolic dysfunction and elevated PVR may contribute to the development of pulmonary arterial hypertension in patients with HFpEF.

## Data Availability

Data will be made available upon reasonable request.
